# *SNHG15* is a bifunctional MYC-regulated noncoding locus encoding a lncRNA that promotes cell proliferation, invasion and drug resistance in colorectal cancer by interacting with AIF

**DOI:** 10.1186/s13046-019-1169-0

**Published:** 2019-04-24

**Authors:** Morvarid Saeinasab, Ahmad Reza Bahrami, Jovanna González, Francesco P. Marchese, Dannys Martinez, Seyed Javad Mowla, Maryam M. Matin, Maite Huarte

**Affiliations:** 1 Department of Biology, Faculty of Science, Ferdowsi University of Mashhad, Mashhad, Iran; 2 Industrial Biotechnology Reasearch Group, Institute of Biotechnology, Ferdowsi University of Mashhad, Mashhad, Iran; 3 Department of Gene Therapy and Regulation of Gene Expression, Center for Applied Medical Research, University of Navarra, Pamplona, Spain; 4 Institute of Health Research of Navarra (IdiSNA), Pamplona, Spain; 5 Department of Molecular Genetics, Faculty of Biological Sciences, Tarbiat Modares University, Tehran, Iran

**Keywords:** *SNHG15*, Colorectal cancer, lncRNA, Survival, Drug resistance, AIF

## Abstract

**Background:**

Thousands of long noncoding RNAs (lncRNAs) are aberrantly expressed in various types of cancers, however our understanding of their role in the disease is still very limited.

**Methods:**

We applied RNAseq analysis from patient-derived data with validation in independent cohort of patients. We followed these studies with gene regulation analysis as well as experimental dissection of the role of the identified lncRNA by multiple in vitro and in vivo methods.

**Results:**

We analyzed RNA-seq data from tumors of 456 CRC patients compared to normal samples, and identified *SNHG15* as a potentially oncogenic lncRNA that encodes a snoRNA in one of its introns. The processed *SNHG15* is overexpressed in CRC tumors and its expression is highly correlated with poor survival of patients. Interestingly, *SNHG15* is more highly expressed in tumors with high levels of *MYC* expression, while MYC protein binds to two E-box motifs on *SNHG15* sequence, indicating that *SNHG15* transcription is directly regulated by the oncogene MYC.

The depletion of *SNHG15* by siRNA or CRISPR-Cas9 inhibits cell proliferation and invasion, decreases colony formation as well as the tumorigenic capacity of CRC cells, whereas its overexpression leads to opposite effects. Gene expression analysis performed upon *SNHG15* inhibition showed changes in multiple relevant genes implicated in cancer progression, including *MYC*, *NRAS*, *BAG3* or *ERBB3*. Several of these genes are functionally related to AIF, a protein that we found to specifically interact with *SNHG15*, suggesting that the *SNHG15* acts, at least in part, by regulating the activity of AIF. Interestingly, ROS levels, which are directly regulated by AIF, show a significant reduction in SNHG15-depleted cells. Moreover, knockdown of *SNHG15* increases the sensitiveness of the cells to 5-FU, while its overexpression renders them more resistant to the chemotherapeutic drug.

**Conclusion:**

Altogether, these results describe an important role of *SNHG15* in promoting colon cancer and mediating drug resistance, suggesting its potential as prognostic marker and target for RNA-based therapies.

**Electronic supplementary material:**

The online version of this article (10.1186/s13046-019-1169-0) contains supplementary material, which is available to authorized users.

## Background

In the past years, advances in sequencing revealed that only 1–2% of the human genome encodes for proteins while the majority of it is transcribed into non-coding RNAs (ncRNAs) [1]. Different classes of ncRNAs are expressed in diverse biological processes and cellular pathways, including small ncRNAs (miRNAs, piRNAs and siRNAs) and long ncRNAs, which have at least 200 nucleotides of length and can be spliced [2–5]. LncRNAs can be classified into various categories based on their position relative to protein-coding genes: (1) intronic, when they are located within genes; (2) intergenic, when they are mapped between different genes and (3) antisense, when they are overlapping with exons of other transcript on the opposite strand [6]. The functional roles of these molecules remain mostly unclear, but recent studies have revealed that they contribute to various cellular processes such as transcription regulation, nuclear architecture, epigenetic regulation, enhancer association (in nucleus), maintenance of mRNA stability, sponging microRNA or regulation of protein translation (in cytoplasm) [7]. Recently, by next-generation sequencing, thousands of lncRNAs have been found to be aberrantly expressed in various types of cancers [8]. While some of them may play oncogenic roles promoting proliferation, invasion and metastasis, others may have a tumor suppressor function, by modulating growth arrest pathways [9–11].

According to global cancer statistics, colorectal cancer (CRC) is the third most common human malignancy and the fourth leading cause of cancer-associated mortality [12, 13]. Therefore, its early diagnosis is an essential requirement. Several studies have reported that some lncRNAs are associated with different stages of CRC [14, 15], indicating that lncRNAs could be a biomarker or target for diagnostic, prognostic and therapeutic applications. However, the contribution of long noncoding RNAs to this type of cancer is still poorly studied.

In the present study, we identify and characterize the oncogenic lncRNA *SNHG15*, which is correlated to survival of CRC patients. Our results describe an important effect of *SNHG15* in cancerous phenotype of CRC cells and its role in drug sensitivity. Moreover, several genes deregulated after *SNHG15* depletion are implicated in cancer initiation, progression and also survival pathways. Altogether, these findings suggest the potential of *SNHG15* as prognostic marker and target for RNA-based therapies.

## Methods

### TCGA analysis

The RNA-seq data of 456 tumor and 41 normal samples were downloaded from TCGA database (https://cancergenome.nih.gov/). The expression of lncRNAs was quantified by Cufflinks v.2.2.1 and lncRNA expression levels were compared between normal tissue and tumor tissue samples.

### Patients

Fresh CRC specimens and their adjacent normal tissues were obtained from 36 CRC patients who underwent surgeries between 2014 and 2016 in Imam-Reza Hospital, Mashhad, Iran. None of patients had received preoperative treatment including radiotherapy or chemotherapy and all of samples were confirmed as colorectal cancer after histopathological examination. The study protocol was approved by the Ethics Committee of Ferdowsi University of Mashhad and all patients were informed with a written for using their tissues. All clinicopathological characteristics of patients are presented in Table 1.

**Table 1:** Clinicopathological characteristics of CRC patients

Clinical parameter	Number	Percentage
Age		
≥ 60	13	36.1
< 60	23	63.9
Gender		
Male	17	47.2
Female	19	52.8
Invasion depth		
T1	2	5.6
T2	4	11.1
T3	29	80.6
T4	1	2.7
TNM stages		
I	3	8.3
II	33	91.7
II & IV	0	0
Lymphatic metastasis		
Yes	18	50
No	18	50

### Cell lines and cell culture

Human colorectal cancer cell lines DLD1, HCT 116, HT-29, LoVo, LS513, SW620 and T84 were cultured in Roswell Park Memorial Institute (RPMI-1640) medium (Gibco) supplemented with 10% fetal bovine serum (FBS, Gibco) and 1% penicillin/streptomycin (Lonza). RKO, SW480 and Caco-2 cells were cultured in Dulbecco’s Modified Eagle Medium (DMEM, Gibco) supplemented with 10% FBS and 1% penicillin/streptomycin. Primary dermal fibroblasts (HDFa) were cultured in High glucose Dulbecco’s Modified Eagle Medium (DMEM, Gibco) supplemented with 15% FBS and 1% penicillin/streptomycin. All cell lines were obtained from American Type Culture Collection (ATCC). Cells were maintained in 5% CO2 humidified-air at 37 °C.

### RNA extraction and qRT-PCR

Total RNAs were isolated from patient specimens and cultured cells using TRIzol Reagent (Invitrogen) following the manufacturer’s instructions. After DNase I (Invitrogen) treatment, cDNAs were synthesized using High-Capacity cDNA Reverse Transcription Kit (Applied Biosystem) with random hexamer primers. qRT-PCRs were performed using SYBR Green reagent (Applied Biosystem). Expression levels of genes were calculated with the comparative cycle threshold (CT) (2-ΔCT and 2-ΔΔCT) method using glyceraldehyde 3-phosphate dehydrogenase (*GAPDH*) as an endogenous control. All primers used in the study are listed in Additional file 1: Table S1.

### Cell transient transfection

All siRNAs for targeting *SNHG15* and *MYC* and negative control siRNA were purchased from Sigma-Aldrich (USA). LoVo and SW620 cells were plated into 6 well plate (150 × 10^3^ cells per well) and transfected with siRNAs at a final concentration of 25 nM for 48 h, using Lipofectamine 2000 (Invitrogen, USA) according to manufacturer’s protocol. The sequence of siRNAs are as follows: *SNHG15*#1 (Sense: 5′-CCUUGAGUCUCAUGUUCAA-3′, Anti-sense: 5′- UUGAACAUGAGACUCAAGG-3′), *SNHG15*#2 (Sense: 5′- GAGCUUACUGUCACAGCAA-3′, Anti-sense: 5′- UUGCUGUGACAGUAAGCUC-3′), *MYC* (Sense: 5′- GGUCAGAGUCUGGAUCACC-3′, Anti-sense: 5′- GGUGAUCCAGACUCUGACC-3′), Ctrl (Sense: 5′- CAGUCGCGUUUGCGACUGGC-3′, Anti-sense: 5′- GCCAGUCGCAAACGCGACUG-3′).

For overexpression of *SNHG15*, we purchased *SNHG15* cDNA sequence (837 bp) was cloned in pDNR-LIB (BC092459; Source Bioscience-UK) and subcloned it into pcDNA3.1 plasmid. Then pcDNA3.1 vectors (empty vector and *SNHG15*) were transfected into HCT 116 and SW480 cells (300 × 10^3^ cells per well) at final concentration 250 ng/mL using Lipofectamine 2000 and subsequent studies were done after 48 h.

### Polysome fractionation

LoVo cells were cultured in 15 cm dishes one day before experiment to reach 80% confluency. The day after, one plate was treated with cycloheximide (100 μg/mL) and another one treated with EDTA (25 mM) to disassemble the polysomes as negative control followed by incubation at 37 °C for 5 min. After removing media and washing 3 times with PBS, cells were harvested by scrapping and transferred to 15-mL tubes for centrifugation (200×*g* for 5 min). Cell pellets were resuspended in 425 μL of a hypotonic buffer [5 mM Tris-HCl (pH 7.5), 1.5 mM KCl, 2.5 mM MgCl_2_ and 1X protease inhibitor cocktail], followed by adding 5 μL of 10 mg/mL CHX or EDTA, 1 μL of 1 M DTT and 100 units of RNase inhibitor and vortexed for 5 s. Then 25 μL of 10% Triton X-100 and 25 μLof 10% sodium deoxycholate were added and vortexed for 5 s again. Cell lysates were centrifuged at 16000×*g* for 7 min at 4 °C and supernatants (~ 500 μl) were loaded onto sucrose gradient. Ultracentrifuge was performed at 33000 rpm for 150 min at 4 °C using Optima L-100 XP Ultracentrifuge (BECKMAN) with SW41Ti rotor. 12 fractions were separated carefully and transferred into 2 mL tubes. 1 mL TRIzol Reagent was added to each fraction and RNA extraction was performed according to manufacturer’s instructions. Expression levels of *SNHG15* in each fraction were quantified by qRT-PCR and normalized relative to the first fraction collected. Also *GAPDH* expression was evaluated as a translated mRNA (positive control).

### Cell proliferation and Colony formation assay

Transfected cells were plated in 96-well plates at a density of 1 × 10^3^ cells per well. Then cell proliferation was evaluated using CellTiter96 Aqueous Non-Radioactive Cell Proliferation Assay (MTS) kit (Promega) every 24 h.

For colony formation assay, transfected cells (0.5 × 10^3^ cells per well) were seeded in a six-well plate. After 10 days, colonies were fixed with 0.5% Glutaraldehyde (Sigma) for 20 min and subsequently washed with PBS for 3 times. Then stained for 30 min with 0.5% crystal violet (Sigma) and the number of colonies was counted in each well.

### Cell-cycle and apoptosis assays

For cell-cycle analysis, transfected cells were harvested after 48 h and stained with propidium iodide. Cell cycle assay was performed in a FACSCalibur flow cytometer (BD Biosciences) and data were analyzed by BD CellQuest and Flow Jo software. For time-line studies, G1/S synchronized cells were generated by double thymidine block procedure. Briefly, cells were grown in medium containing 2 mM thymidine for 16 h. Then cultured in normal medium for 9 h followed by 16 h incubation in presence of 2 mM thymidine again.

Apoptosis assay was performed by Annexin V and 7-AAD staining using Apoptosis Detection Kit I (BD Biosciences) according to manufacturer’s instructions and detected by FACSCalibur flowcytometer.

### Transwell invasion assay

For invasion assay, the upper side of 8-μm pore-size transwell inserts (Corning) were precoated with type I rat tail collagen (Croning) and then Matrigel (BD, Biosciences) was diluted with PBS (3 mg/mL) and polymerized at 37 °C for 2 h. 48 h after transfection, 10^5^ CRC cells (LoVo or HCT116) in 100 μL of medium containing 1% FBS, were plated onto the upper side of inserts and after 4 h, 300 μL of medium containing 10% FBS was added to the lower chamber to induce cell attraction. Plates were incubated at 37 °C for 48. Then cells were fixed in 4% formaldehyde for 20 min and non-invading cells on the upper side of the insert were removed with cotton swabs. The lower part of the insert was stained with 0.1% crystal violet (Sigma). Images were captured from four fields in each well using the Leica DMIL LED inverted microscope (Leica Microsystems) and the numbers of invasive cells were counted from five random fields in each image. Experiments were performed independently at least three times.

### Mouse xenograft experiments

Female BALB/c-Rag2/−IL2cc/immunodeficient mice aged 6–7 weeks were used in this study. The study was performed under specific pathogen-free conditions at Center of Medical Application (CIMA) University of Navarra, Spain. For each mice, 1 × 10^6^ *SNHG15* overexpressing HCT 116 and HCT116 transfected with an empty vector were resuspended in 100 μL PBS and mixed with the same amount of Matrigel to inject subcutaneously into the hind limb. Tumor size was measured externally every 4 days using a precision caliper for a total period of 28 days. Tumor volume (V) was estimated using the following formula: V = π/6 × width^2^ × length. The tumor weight was measured on the last day after removal.

For LoVo cells, after CRISPR-Cas9 editing, 4× 10^6^ cells were injected per mice and tumor size was measured every 4 days. After 48 days, tumors were dissected out and their weight were measured.

### CRISPR-Cas9 editing

Two single-guide RNAs (sgRNAs) were designed to delete the region between exon 3 to 5 of *SNHG15* using a tool from the Zhang Lab (http://crispr.mit.edu/). Oligonucleotides to clone the guide RNA (Additional file 2: Table S2) were then annealed and cloned into pX330 vector containing CAS9 [16] and subsequently co-transfected with GFP expressing plasmid (pmax-GFP) into LoVo cells. GFP positive cells were sorted in 96 well plate by BD FACSAria IIu cytometer. After single cells reached confluency, genomic DNA was extracted using QuickExtract reagent (Epicentre) and PCR was performed using a pair of primers flanking the depleted region and positive clones were identified by PCR product length. Furthermore, RNA was extracted to perform qRT-PCR by specific primer (Primer pair 1 and 2) to validate deletion of aimed region. After selecting clones (WT3, CL10 and CL83), subsequent experiment (cell proliferation, colony formation, cell cycle, apoptosis assay and tumor formation) were performed as described before.

### Nuclear-cytoplasmic fractionation

Cells were cultured in 10 cm dishes and harvested into two tubes for subcellular fractions and whole-cell extraction. After centrifugation at 1000×*g* for 5 min at 4 °C, cell pellets were resuspended in 500 μL of lysis buffer (10 mM Tris-HCl, pH 7.5, 140 mM NaCl, 1.5 mM MgCl_2_, 0.05% IGEPAL supplemented with RNasin 10 U/mL and protease inhibitor cocktail) and incubated on ice for 10 min. Then TRIzol Reagent was added to one tube containing cell lysate to extract total RNA. For obtaining nuclear and cytoplasmic fractions, 500 μL of lysis buffer containing sucrose was added into a clean tube and cell lysate was added to this tube without mixing the two phases. After 10 min centrifugation at 12000×*g* and 4 °C, around 500 μL of the upper phase was collected as cytoplasmic fraction. Remaining pellet was resuspended in 500 μL of lysis buffer as nuclear fraction and finally cytoplasmic and nuclear RNAs were extracted using TRIzol Reagent.

### RNA pull-down

RNA pull-down was performed as described previously [17]. Briefly, biotinylated RNA of *SNHG15* was generated in vitro and incubated with protein lysate of LoVo cells and then streptavidin magnetic beads. Interacting proteins were loaded in a NuPAGE Novex 4–12% bis-Tris gel (Invitrogen) and stained with SilverQuest Silver Staining Kit (ThermoFisher). One differential band was seen and sent for mass spectrometry to Taplin Mass Spectrometry Facility (Harvard Medical School; USA).

### Western blot

Cells were harvested and lysed in RIPA buffer (150 mM NaCl, 0.5% sodium deoxycholate (Sigma), 0.1% SDS (Sigma), 50 mM Tris–HCl (pH 7.4), 150 mM NaCl (Sigma)) containing protease Inhibitor Cocktail (Roche Applied Science) for 15 min on ice. Cell lysate was centrifuged at 16000×*g* at 4 °C for 15 min and protein concentration was measured by Pierce BCA Protein assay kit (ThermoFisher). Equal amounts of proteins were loaded on a 10% SDS-PAG and after separation by electrophoresis, transferred onto nitrocellulose membranes (Bio-Rad). Membranes were then blocked in 5% non-fat milk for 1 h at RT and immunoblotted by overnight incubation at 4 °C with the indicated anti-AIF primary antibody (1:1000; sc-13,116; Santa Cruz). After three washes with PBS-Tween (0.1%), the membranes were incubated with anti-mouse secondary antibody: anti-IgG (1:2000; sc-2025; Cell Signaling) for 1 h at RT. Finally, the protein bands were visualized using an Enhanced Chemiluminescence Detection kit (Perkin Elmer, Waltham, MA, USA).

### RNA immunoprecipitation (RIP)

LoVo cells were cultured in 10 cm dishes and then harvested to prepare cell lysate by RIPA buffer. After centrifugation, cytoplasmic fraction was collected and pre-cleared with Protein G beads (Invitrogen). Five percent of samples were used as input and remaining were divided to two parts and incubated with AIF monoclonal antibody or IgG overnight at 4 °C. RNAs bound to specific proteins, were separated by Dynabeads protein G beads (Invitrogen) and extracted by TRIzol. *SNHG15* RNA levels were quantified by qRT-PCR using 3 pairs of *SNHG15* primers. *MALAT-1*, *U6*, *GAPDH* and *HPRT* were used as negative controls and the data were presented to the value obtained from IgG.

### Immunofluorescence

A density of 75 × 10^3^ LoVo cells were seeded on coverslips placed in the bottom of each well in 12 well plates. 48 h after inhibition by siRNAs, cells were fixed in 4% PFA solution for 30 min at RT and then washed with washing buffer. Non-specific binding was blocked using block solution containing 10% FBS. Cells were incubated in anti-AIF prepared in block solution (1:250; sc-13,116; Santa Cruz) for 30 min at RT. Then washed two times with washing buffer, followed by 30 min incubation in secondary Alexa fluor 488 donkey anti-mouse IgG (A-21202, Thermo Fisher). Coverslips were mounted on slides using mounting solution. All images were captured using LSM 800 (Zeiss, Jena, Germany) inverted confocal microscope equipped with a 63x Plan-Apochromat objective (NA1.4 oil).

### ROS assay

LoVo cells were transfected with combination of two si-*SNHG15* and si-control. After 48 h, cells were collected and cell lysates were prepared. Cellular ROS level were measured by OxiSelect in vitro ROS/RNS assay kit (Cell biolabs) according to the manufacturer’s instructions, using fluorescence plate reader. Results are shown as change in relative fluorescence unit (RFU).

### Chemotherapy sensitivity assay

24 h after transfection of LoVo or HCT 116 cells, 5 × 10^3^ cells were plated in each well of 96-plates. Plates were incubated for another 24 h and then treated with 0 to 50 μg/mL of 5-FU (Sigma). Cells proliferation was determined using CellTiter96 Aqueous Non-Radioactive Cell Proliferation Assay (MTS) kit (Promega®) every 24 h for upto 3 days. Viability of the cells was determined by the following equation: (cells treated with 5-FU Abs/ Untreated cells Abs) × 100.

### RNA sequencing

LoVo cells were transfected with combination of two siRNA and control si-RNA. RNA was extracted by Maxwell 16 LEV simply RNA kit (Promega) from three biological replicates and the quality of them, were assessed by High sensitivity RNA ScreenTape system (Agilent Technologies). Library preparation was performed with the TruSeq Stranded mRNA kit (Illumina). Libraries were then sequenced on an Illumina NextSeq (75 bp paired-end). Sequenced reads were aligned using bowtie2 (against hg19) and the differential gene expression analysis was carried out with DeSeq2. Biological knowledge extracted by using Ingenuty Pathway Analysis (QIAGEN Inc., https://www.qiagenbioinformatics.com/products/ingeuity-pathway-analysis).

### Statistical analysis

All data were analyzed using GraphPad Prism software (GraphPad Software, version 5.01, CA, USA). Two-tailed student’s t-test was used to analyze normal distributed data and differences were considered significant at *p* < 0.05.

## Results

### *SNHG15* is upregulated in colorectal cancer and is highly correlated with poor survival

**Fig. 1:**
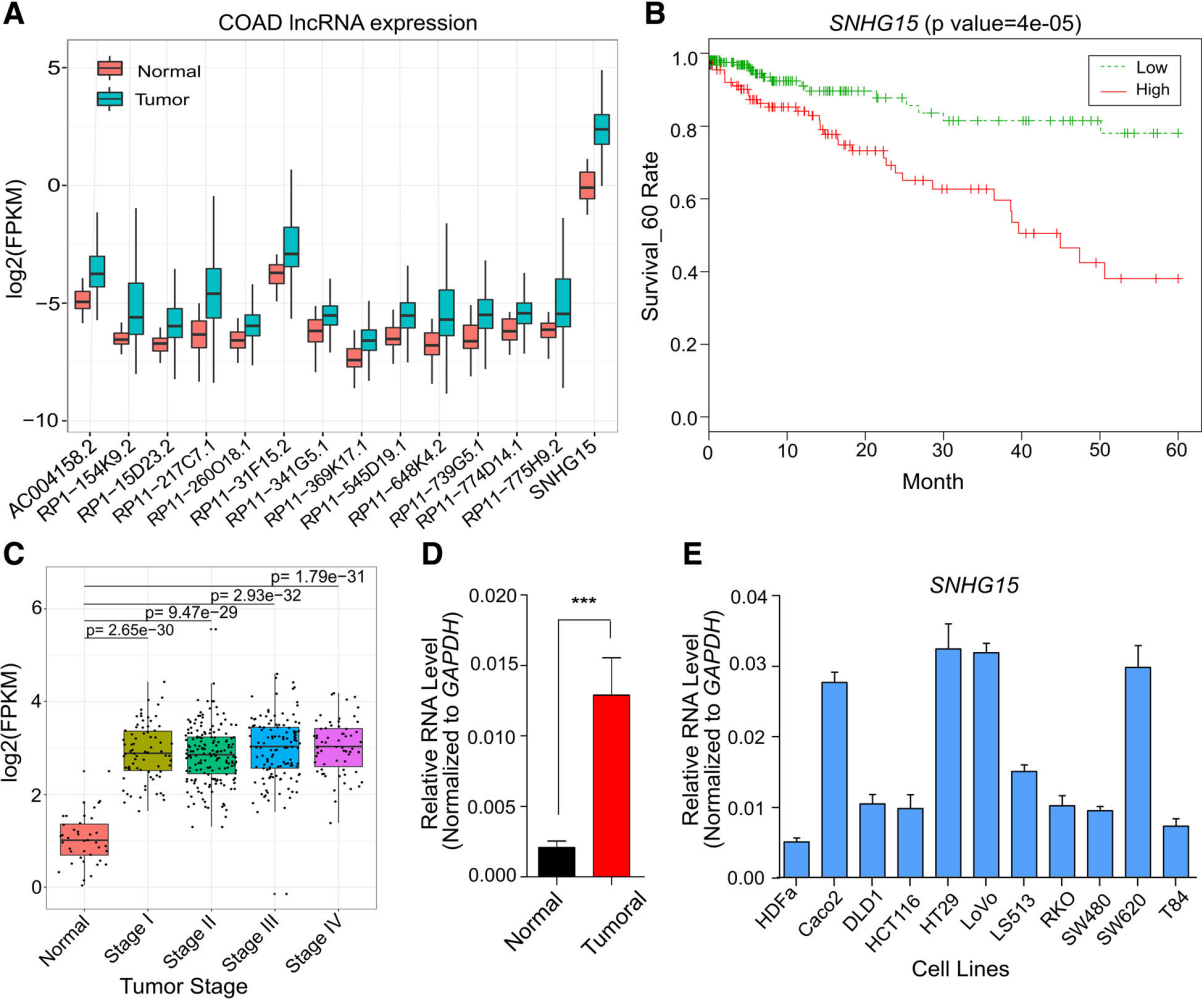
**a** Expression of candidate lncRNAs deregulated in CRC tumors compared to normal samples and with higher expression significantly correlated with decreased survival of patients analyzed by RNA-seq from The Cancer Genome Atlas (TCGA). *P* values were calculated using Wilcoxon signed rank test. **b** Kaplan–Meier analyses of the correlations between *SNHG15* expression level and overall survival of 450 patients with CRC (TCGA). **c** *SNHG15* expression levels in different stages of CRC compared with normal tissues (TCGA). **d** Relative expression level of *SNHG15* in 36 Iranian CRC patients compared with corresponding adjacent tissue. *P*-value is calculated by two-tailed Student’s t-test. **e** Expression levels of *SNHG15* in a panel of CRC cell lines in comparison with HDFa cells as a normal cell line. Data are shown as mean ± SD

To characterize lncRNAs deregulated in CRC and correlated with survival, we profiled their expression in Tumor Cancer Genome Atlas (TCGA) cohort of 456 tumoral and 41 normal tissue samples. We found 14 lncRNAs as the most significantly deregulated transcripts (Fig. 1a, Additional file 3: Table S3), for which their upregulation was related to a significant decrease in survival of CRC patients (Table S3). Among them, we focused on *SNHG15* due to its highly significant upregulation in tumors (*p* value =7.5e-23) and also the highest correlation with poor survival of patients (*p* value = 4e-5) (Fig. 1b). More investigations in this cohort of tumors containing different stages of CRC revealed that although there is a significant upregulation of *SNHG15* expression in tumors versus normal samples, there is no obvious difference among CRC patients at various stages (Fig. 1c). These results suggest that *SNHG15* upregulation is an early event in colorectal cancer promotion and its expression is maintained at high levels until last stage.

To confirm this observation, we obtained 36 fresh colorectal cancer tumors and their adjacent normal tissues immediately after surgery from Iranian CRC patients (Table 1). *SNHG15* expression was examined by qRT-PCR and its upregulation was observed in tumoral samples (Fig. 1d, *p* < 0.001). Moreover, we profiled *SNHG15* expression in ten CRC cell lines (Caco-2, DLD-1, HCT 116, HT-29, LoVo, LS513, RKO, SW480, SW620 and T84) and found this lncRNA ubiquitously expressed in all tested CRC cell lines and with higher levels compared to the non-cancerous HDFa cell line (Fig. 1e).

### *SNHG15* expression is regulated in the cell cycle and transcriptionally controlled by MYC

**Fig. 2:**
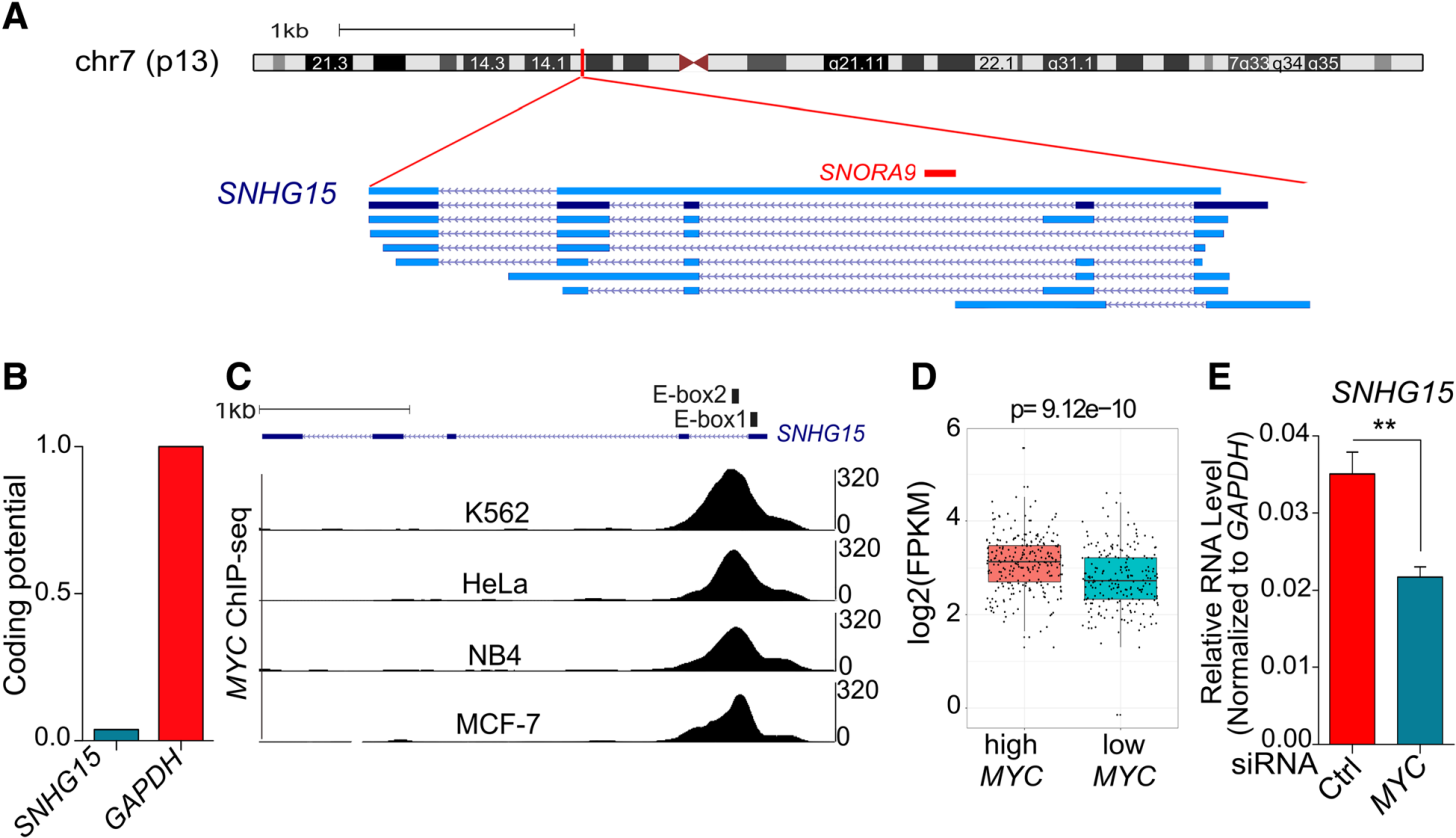
**a** Genomic locus of *SNHG15* and its different transcript variants annotated in Gencode V29. The most highly expressed variant is shown in dark blue. **b** Coding potential of *SNHG15* in comparison with *GAPDH* (as a coding-protein gene) determined with coding potential assessment tool (CPAT). **c** Location of the E-box biding motifs in the *SNHG15* sequence and MYC binding to these regions confirmed by ChIP-seq in different cancerous cell lines. **d** Expression of *SNHG15* in CRC tumors with high or low *MYC* expression (TCGA). Significance was determined by unpaired student’s t-test (*p* value < 0.001). **e** *SNHG15* RNA level after depletion of *MYC* in LoVo cells. *p* value is calculated by two-tailed Student’s t-test (*p* value < 0.01)

*SNHG15* (small nucleolar RNA host gene 15) is located on chromosome 7 and composed of 5 exons. According to GENCODE V29 annotation, it can be spliced into different isoforms, although isoform 2 is the most abundant [18]. Interestingly, *SNORA9* (small nucleolar RNA, H/ACA box 9) is encoded in intron 2 of *SNHG15* (Fig. 2a). *SNORA9* also named ACA9 is a member of H/ACA pseudouridylation guide RNA machinery, which contributes to pseudouridine synthesis in snRNAs and rRNAs [19]. Coding potential analysis revealed that *SNHG15* sequence does not have ability to code for proteins (Fig. 2b). In agreement with this, polysome fractionation methods revealed that *SNHG15* is not associated with polysomes (Additional file 4: Figure S1).

Previous studies revealed that there are two E-box (CACGTG) binding motifs for transcription factor MYC on the first exon and first intron of *SNHG15* [20]. We therefore analyzed ChIP-seq data from ENCODE and confirmed that MYC is bound to these boxes in different cancerous cell lines (Fig. 2c).

In order to investigate the transcriptional regulation of *SNHG15* by MYC, we analyzed colorectal adenocarcinoma RNA-seq data from TCGA, finding that *SNHG15* is significantly upregulated in the tumors with high level of *MYC* expression (Fig. 2d). In agreement with this observation, the depletion of *MYC* in LoVo CRC cell line resulted in a significant decrease in the level of *SNHG15* (Fig. 2e). These results indicate that *SNHG15* transcription is controlled by MYC.

Since MYC is well known to regulate cell cycle, we investigated expression of *SNHG15* during cell cycle. To do this, we obtained G1/S synchronized cells by double thymidine block procedure and collected the cells at different time points after block release. Results showed that *SNHG15* expression is regulated during the cell cycle, with an increased expression of *SNHG15* in G2/M phase (Additional file 5: Figure S2). Together, these data suggest that *SNHG15* is transcriptionally regulated by MYC in a cell-cycle dependent manner.

### *SNHG15* deregulation has strong effects on proliferation, invasion and tumor formation abilities of CRC cells

**Fig. 3:**
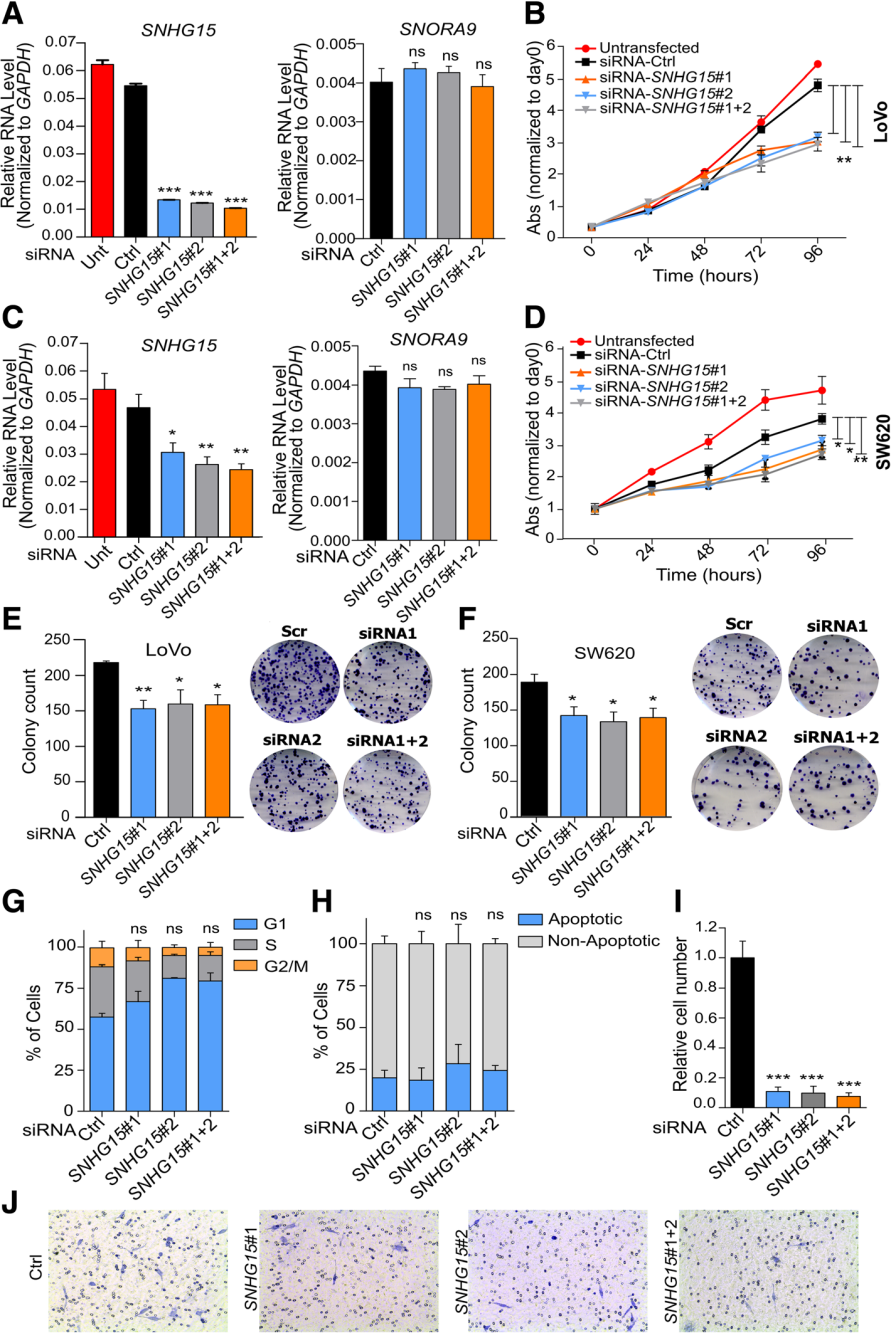
**a** *SNHG15* and *SNORA9* RNA level after knockdown with the two siRNAs separately or in combination in LoVo cells. **b** Cell proliferation measured by MTS assay after inhibition of *SNHG15* in LoVo cells. **c** *SNHG15* and *SNORA9* expression levels in SW620 cells after inhibition of *SNHG15* with siRNAs. **d** Proliferation capacity of SW620 after *SNHG15* knockdown. **e** The results of colony formation assay performed quantified after 10 days of inhibition in LoVo cell line and (**f**) in SW620 cell line. **g** Cell-cycle phase distribution of LoVo cells determined by propidium iodide-staining (**h**) Percentage of apoptotic LoVo cells after staining with annexin V and 7-AAD (**i**) Number of LoVo cells invading through the membrane under the indicated conditions in the transwell assay. **j** Invading LoVo cells stained on transwell chambers after 48 h in each condition. The statistical analysis is performed by two-tailed Student’s t-test and graphs shows mean ± SEM of values (*p < 0.05, **p < 0.01, ***p < 0.001)

To investigate *SNHG15* function in CRC, we designed two siRNAs to knock it down, and transfected LoVo and SW620 cells with each one of them individually or a combination of both. qRT-PCR analysis showed that 48 h after transfection, *SNHG15* transcript was significantly reduced, while the expression level of *SNORA9*, which is located in one of its introns, was not changed (Fig. 3a and c). These results confirmed that the designed siRNAs target *SNHG15* exons after splicing, leaving intact *SNORA9*, and allowing studying *SNHG15* function independently of *SNORA9*. Further investigation showed that the knockdown of *SNHG15* significantly inhibited cell proliferation (Fig. 3b and d) and colony formation capacity of these cells (Fig. 3e-f). However, downregulation of *SNHG15* did not significantly influence the cell cycle profile or the percentage of apoptotic cells (Fig. 3g-h). On the other hand, the invasion capacity of the cells was significantly decreased after *SNHG15* inhibition, as quantified by transwell assays (Fig. 3i-j).

**Fig. 4:**
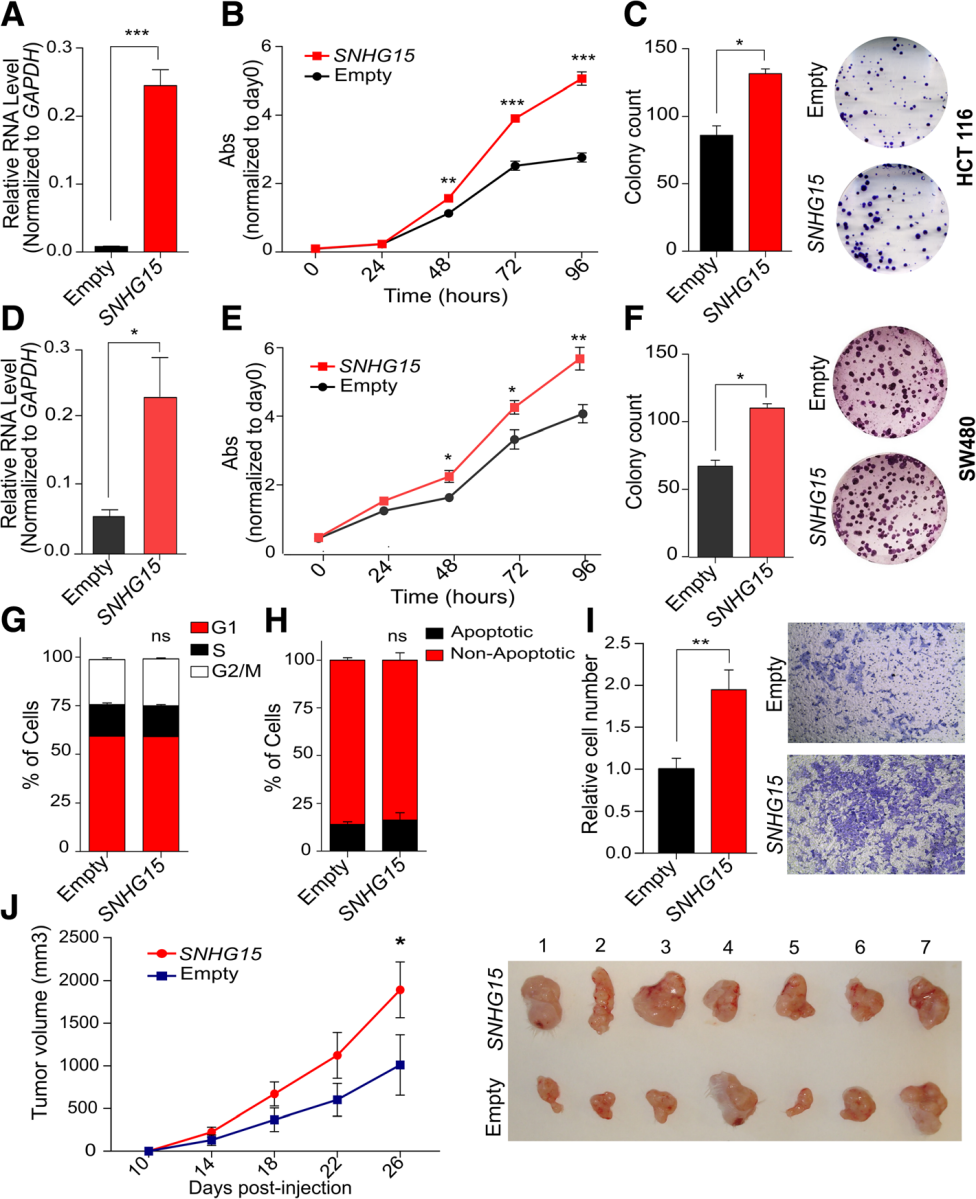
**a** Level of *SNHG15* after overexpression in HCT116 cells determined by qRT-PCR (**b**) Change in proliferation ability of HCT116 cells after overexpression of *SNHG15* in comparison with control. **c** Colony formation ability in overexpressing cells comparing to control cells. **d** *SNHG15* RNA level after Transfection SW480 cells with SNHG15-vector via Empty vector. **e** Change in cell proliferation ability after *SNHG15* overexpression in SW480 cell line. **f** Results of colony formation assay after overexpressing *SNHG15* in SW480 cells. **g** Cell cycle analysis in HCT 116 cells after overexpression of *SNHG15* using propidium iodide staining. **h** Percentage of apoptotic HCT 116 cells determined by annexin V and 7-AAD staining. **i** Invasion ability of HCT116 cells after overexpression of *SNHG15*. **j** Tumor formation in immunodeficient mice of the same cell lines (*n* = 7). *P* value is calculated by two-tailed Student’s t-test and graphs shows mean ± SEM of values (*p < 0.05, **p < 0.01, ***p < 0.001)

We subsequently investigated the effects of *SNHG15* overexpression on cell growth, invasion and tumor formation capacity. To do so, *SNHG15* cDNA sequence (837 bp) was cloned and expressed transiently in HCT 116 and SW480 cells, which express *SNHG15* at lower levels compared to LoVo and SW620 cells. qRT-PCR analysis indicated a significant increase in *SNHG15* RNA levels relative to the control cells transfected with the empty vector (Fig. 4a and d). MTS assay showed that the enforced expression of *SNHG15* led to a significant increase in cell proliferation (Fig. 4b and e). Colony formation assay also indicated that *SNHG15*-overexpressing cells not only could form more colonies but also of larger size (Fig. 4c and f). Consistently with the phenotype observed upon depletion, *SNHG15* overexpression did not influence cell cycle or apoptosis in HCT 116 cells (Fig. 4g-h). In addition, the overexpression of *SNHG15* in HCT 116 cells increased their invasion capacity (Fig. 4i).

To further explore the role of *SNHG15* in the tumorigenicity of CRC cells, *SNHG15*-overexpressing and control HCT 116 cells were injected into immunodeficient mice, and the tumor size was measured every 4 days. As shown in Fig. 4j, tumors grew faster in cells overexpressed *SNHG15*, and larger and heavier tumors were formed by these cells after 4 weeks.

**Fig. 5:**
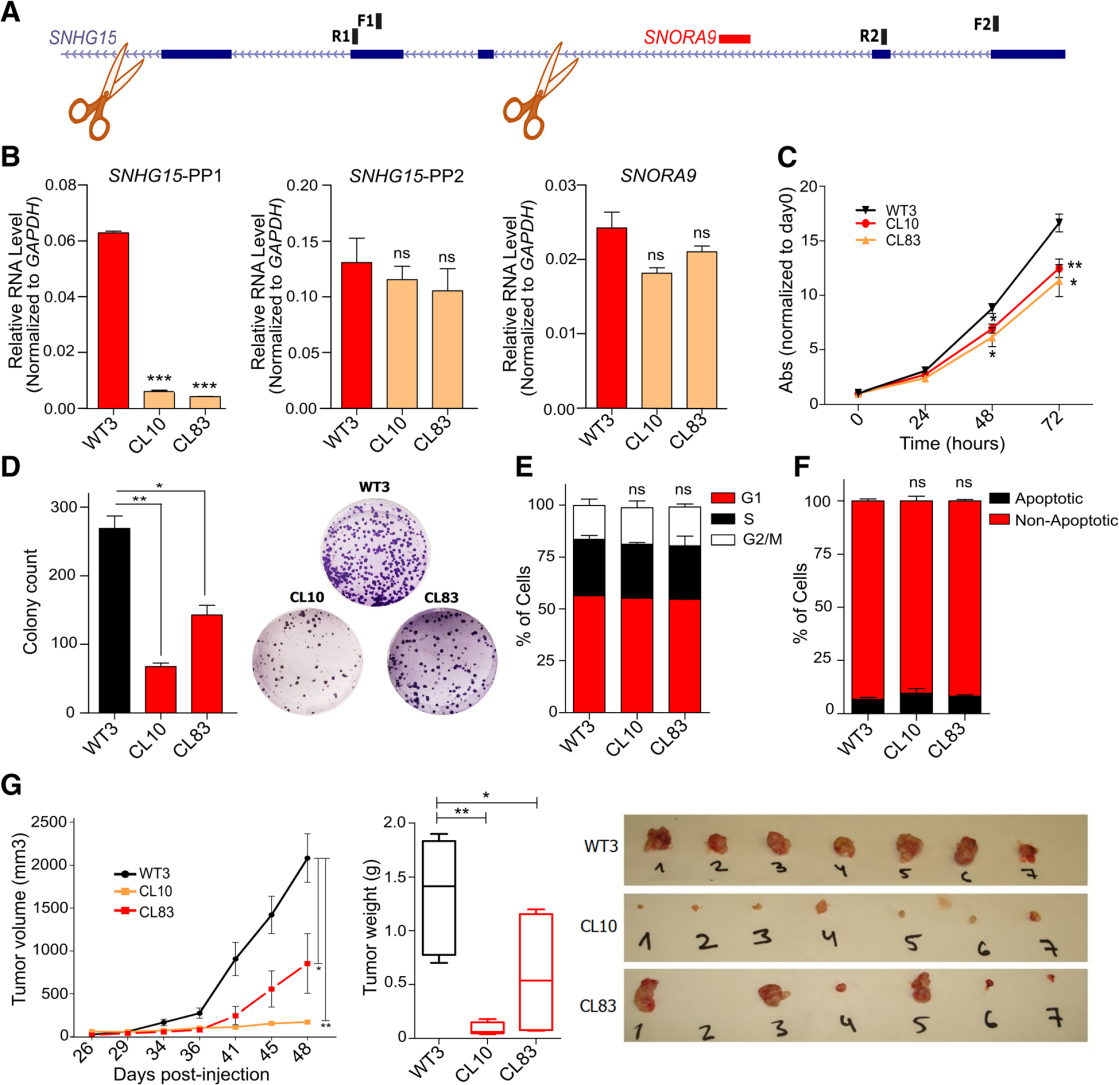
**a** Schematic representation of CRISPR/Cas9 technology to delete the region between exons 3 to 5 of *SNHG15* and annealing sites of primers to detect deleted and non-deleted regions on RNA. **b** Relative RNA level for deleted, non-deleted region and *SNORA9* from wild type (no. 3) and homozygote clones (no. 10 and no. 83) with the different primer sets. **c** Cell proliferation, **d** colony formation ability after knockout of *SNHG15*. **e** Cell cycle profile and (**f**) Percentage of apototic cells. **g** Tumor formation capacity after deletion of exons 3 to 5 of *SNHG15* in LoVo cells. The statistical analysis is performed by two-tailed Student’s t-test and graphs shows mean ± SEM of values (*p < 0.05, **p < 0.01, ***p < 0.001)

In order to confirm our results and exclude possible off target effects of the siRNAs, we knocked-out *SNHG15* by CRISPR-Cas9 system in LoVo cells. Two guide RNAs (gRNAs) were designed to delete a region of *SNHG15* of around 1400 bp without affecting *SNORA9* sequence (Fig. 5a). Several clones were obtained with homozygote deletion. After screening of the clones, we chose two independent clones with deletion in the aimed region confirmed by qRT-PCR with two pairs of primer designed specifically for deleted and non-deleted region. Of note, they no changes in *SNORA9* expression were observed (Fig. 5b). The experimental characterization of these two clones showed their low proliferation and colony formation capacity (Fig. 5c, d), while didn’t show significant changes in cell cycle profile and percentage of apoptotic cells (Fig. 5e-f). Moreover, xenograft mice model experiments confirmed our previous data and revealed that the tumors formed by knock-out cells were smaller and lighter than those formed by the wild type cells (Fig. 5g). Together, these results suggest that *SNHG15* promotes the oncogenic capacity of CRC cells.

### *SNHG15* depletion in CRC cells affects the expression of genes with roles in cell proliferation, migration and survival

**Fig. 6:**
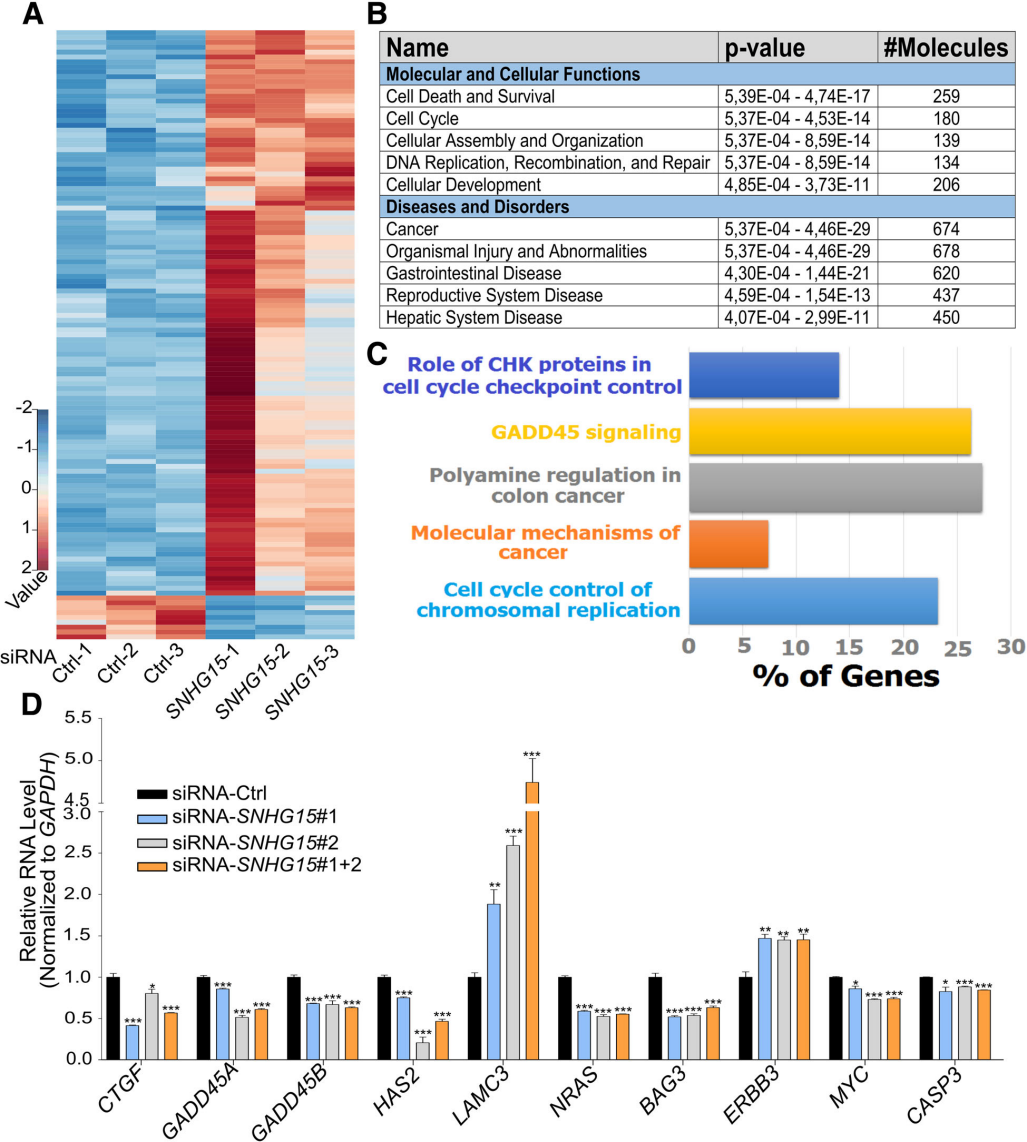
**a** Heatmap representation of genes deregulated after *SNHG15* depletion by siRNA in LoVo cells (*p* value < 0.01). **b** Molecular and cellular functions and diseases associated with these genes. *P* values are the min and max *p* values of the enriched categories within a general category that appears in the data Table. **c** Top canonical pathways changed after depletion of *SNHG15* recognized by IPA*.* **d** qRT-PCR analysis for RNA-seq validation on the genes selected from RNA-seq and pathway analysies. *P* value is calculated by two-tailed Student’s t-test and graphs shows mean ± SEM of values (*p < 0.05, **p < 0.01, ***p < 0.001)

To determine the biological processes and pathways regulated by *SNHG15*, a gene expression analysis by RNA-seq was performed in LoVo cells after transfection with combination of two siRNAs or a negative control siRNA. Our results revealed an elevated number of genes significantly deregulated upon *SNHG15* depletion (Fig. 6a – Additional file 6: Table S4). Among 766 genes with a significant change of expression (FDR < 0.05), 372 genes were upregulated and 394 genes were downregulated. In order to obtain more information about *SNHG15* function, Ingenuity Pathway Analysis (IPA) was performed, showing that the genes affected by *SNHG15* knockdown are preferentially associated to cancer as well as cell death and survival (Fig. 6b). Interestingly, more detailed canonical pathway analysis showed that these genes contribute to some important molecular pathways and mechanisms of cancer, including polyamine regulation in colon cancer, GADD45 signaling, chromosomal replication during cell cycle and role of CHK protein in cell cycle checkpoint control (Fig. 6c). We chose 20 candidates among the genes detected by the RNA-seq and pathway analyses to validate the changes in their expression caused by *SNHG15* depletion. Results showed that *CTGF*, *GADD45A*, *GADD45B*, *HAS2*, *LAMC3*, *NRAS*, *BAG3*, *ERBB3*, *MYC* and *CASP3* were deregulated after *SNHG15* inhibition with each individual siRNA or by the combination of them (Fig. 6c), confirming the effect of *SNHG15* in the regulation of these relevant genes.

### *SNHG15* interacts with AIF in the cytoplasm and contributes to the resistance to stress

**Fig. 7:**
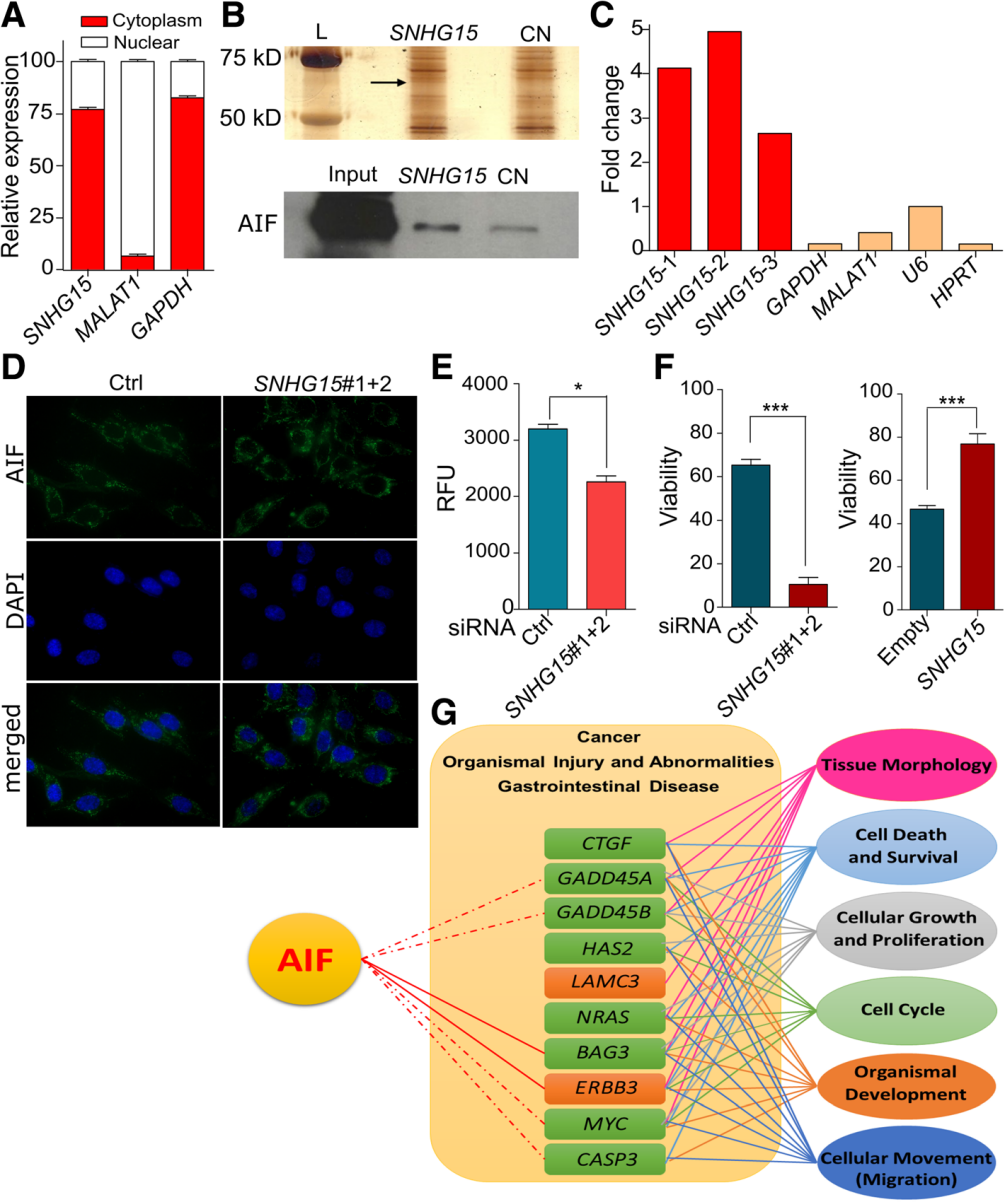
**a** RNA levels of *SNHG15* and control RNAs in nuclear and cytoplasmic fractions measured by qRT-PCR. The statistical analysis is performed by two-tailed Student’s t-test **b** Top: Silver stained gel with the proteins retained in the RNA pull-down experiment by *SNHG15* and linc-p21 as negative control (CN), the differential band analyzed by mass spectrometry is indicated by an arrowhead; bottom: detection of the AIF protein by western blot in a replicate of the RNA pull-down experiment. **c** RNA immunoprecipitation using AIF monoclonal antibody and followed by qRT-PCR using three different primer pairs for *SNHG15*, and *MALAT-1*, *U6*, *GAPDH* and *HPRT* as negative controls. **d** AIF localization after *SNHG15* inhibition using immunofluorescence antibody. Nuclei were stained with DAPI. **e** ROS levels of in LoVo cells transfected with the indicate siRNAs (*p* value< 0.05). **f** Cell viability determined by MTS of CRC cells (LoVo and HCT 116) after depletion or overexpression of *SNHG15* treated with 8 μg/mL for 48 h (*p* value< 0.001). **g** Connection between AIF and the genes validated with most important pathway recognized. The statistical analysis is performed by two-tailed Student’s t-test and graphs shows mean ± SEM of values

The gene expression analysis performed reflected the physiological changes caused by *SNHG15* downregulation in CRC cells in terms of proliferation and tumorigenicity. However the analysis did not provide insights into the mechanism by which *SNHG15* affects the biology of the tumor cells. To investigate the mechanism by which *SNHG15* regulates CRC proliferation, we first set to determine its subcellular localization. Nuclear-cytoplasmic fractionation in LoVo cells showed that this lncRNA is mainly cytoplasmic (Fig. 7a). We hypothesized that *SNHG15* interacts with cytoplasmic proteins to carry out its functions, so we focused on identifying specific physical interactions with *SNHG15*. To that end, we performed RNA pull-down using in vitro transcribed *SNHG15* RNA or an unrelated RNA of similar length (murine linc-p21) as control. The RNAs were incubated with cytoplasmic extract of LoVo cells and mass spectrometry (MS) was performed on the differential band found on the retained proteins (Fig. 7b, upper panel). Apoptosis Induced Factor (AIF) was identified as a protein bound to *SNHG15* with 8 unique peptides but absent in the control RNA pull-down. AIF is a bifunctional protein that exhibits distinct roles based on its subcellular localization. After translation in the cytosol AIF is transported to the mitochondria, where it acts as an NADH oxidase to generate O_2_^−^ and subsequently H_2_O_2_ [21]. In consequence, AIF has an effect on reactive oxygen species (ROS) levels with a strong impact in various cellular stress and survival pathways [22, 23]. On the other hand, upon apoptotic stimulus AIF can be cleaved and translocated to the nucleus, where it binds to DNA and promotes chromatin condensation and DNA fragmentation, which are necessary for the apoptosis program [24]. Interestingly, the molecular weight of the isoform that we found as interacting with *SNHG15* is around 67 kDa (full length protein), indicating that *SNHG15* binds to the precursor AIF.

The interaction between *SNHG15* and AIF was further validated by western blot using specific antibody against AIF (Fig. 7b). Moreover, it was confirmed by RNA immunoprecipitation (RIP) with an antibody able to specifically immunoprecipitate the endogenous AIF (Fig. 7c). To further investigate the functional relationship between *SNHG15* and AIF, immunofluorescence studies were performed showing that inhibition of *SNHG15* did not change AIF subcellular localization from cytoplasm to nucleus (Fig. 7d), suggesting that *SNHG15* is not implicated in the pro-apoptotic function of AIF.

Based on these observations and the phenotype observed in the cells upon *SNHG15* inhibiton, we hypothesized that *SNHG15* binding to AIF does not influence AIF role in apoptosis, but may affect the other mechanism in which the protein has been involved, i.e. respiratory chain and stress response [22, 23]. To test this hypothesis, we measured ROS levels in *SNHG15*-depleted cells in comparison to controls. As shown in Fig. 7e, after depletion of *SNHG15*, ROS levels resulted in a significant reduction. Since enhancement of ROS is known to prevent cellular toxicity by neutralizing chemical stresses [22, 23], we examined whether the expression of *SNHG15* had an influence on survival of CRC cells after treatment with 5-FU as chemical stress. To do this, we depleted or overexpressed *SNHG15* in CRC cells (LoVo or HCT 116) and exposed them to different concentrations of 5-FU (0 to 50 μg/mL). After 48 h of drug treatment, the results on proliferation assay showed that *SNHG15* levels had a significant influence on the response of CRC to 5-FU at 8 μg/mL. At this concentration, *SNHG15*-depleted cells were more sensitive to 5-FU and their viability was lower compared to control cells. On the other hand, *SNHG15*-overexpressing cells showed more resistance and higher survival to the drug treatment than control cells (Fig. 7f). These results suggest that the increased levels of *SNHG15* are related with the capacity of CRC cells to cope with the cytotoxic stress caused by 5-FU, which could be mediated by its interaction with AIF. In agreement with these findings, pathway analysis on the genes affected by *SNHG15* knockdown revealed that many of them are functionally related to AIF. Moreover, the role of this group of genes in many important cellular mechanisms such as tissue morphology, cell death and survival, cellular growth and proliferation, cell cycle, organismal development and cellular movement (migration) was confirmed. It was also revealed that these genes contributed in some disorder like cancer, gastrointestinal disease, organismal injury and abnormalities (Fig. 7g).

## Discussion

Since colorectal cancer is the third most common human malignancy worldwide [13], many researchers have focused on the characterization of CRC-related lncRNAs as new biomarkers for diagnosis or targeted therapy of this disease. Some of these important lncRNAs include *CCAT1*, *H19*, *HOTAIR*, *MALAT1*, *UCA1* and *PTENP1* [14, 25]. In this study, we searched for lncRNAs strongly associated with poor prognosis of CRC. *SNHG15* was identified as an oncogenic lncRNA whose upregulation was related to poor survival of CRC patients. Interestingly, the classification of colorectal adenocarcinoma TCGA samples relative to *MYC* expression showed that *SNHG15* is upregulated in the samples with high levels of *MYC* expression. More investigation confirmed that MYC has two binding sites (E-box) on *SNHG15* sequence and bound them in different cancerous cell lines. Furthermore, its inhibition by siRNA, led to decrease *SNHG15* level in CRC cell line and confirmed that *SNHG15* is transcriptionally regulated by MYC. On the other hand, RNA-seq results showed significant reduction of *MYC* transcript after depletion of *SNHG15*. These finding suggest a feedback loop between *SNHG15* and *MYC* expression that introduce *SNHG15* as an additional component of the pro-proliferative network activated by this oncogenic transcription factor. Consistently with this notion, the depletion of *SNHG15* by different experimental methods leads to decreased cell proliferation, while its enforced expression promotes cell proliferation and clonogenicity.

Long non-coding RNAs have been involved in a variety of mechanisms that can take place in different cellular compartments. Although some studies have addressed the role of *SNHG15* in the nucleus [18], in LoVo cells *SNHG15* is mainly localized in the cytoplasm, suggesting that it has a role in this cellular compartment. To elucidate the role of *SNHG15*, we searched for proteins with specific physical interactions with *SNHG15*, identifying AIF as associated with *SNHG15*. AIF mRNA is translated in the cytoplasm into a 613-amino acid precursor (67 kDa) that is transported to mitochondria via mitochondrial localization signal (MLS). After being imported into the intermembrane space, the first 34 amino acids are removed and the 62 kDa mature AIF (AIFmit) is generated to contribute to the respiratory chain as an NADH oxidase [21, 26]. If cells receive an apoptotic stimulus, a different cleavage occurs in AIFmit by calpain or cathepsin, and a 57 kDa AIF is formed to induce DNA condensation and DNA fragmentation in the nucleus [27–29].

It has been shown that AIF maintains the transformed state of CRC cells via its NADH oxidase activity and cells show more apoptosis sensitivity as a result of AIF knockout and decreases in ROS level. In other words, AIF helps to neutralize chemical stress, and increased protein level of AIF leads to enhancement of ROS to prevent cellular toxicity [22, 23]. Interestingly, we did not observe significant changes in the number of apoptotic cells after dysregulation of *SNHG15*. In addition, inhibition of *SNHG15* did not induce translocation of AIF into nucleus after 48 h. These data suggest that under these experimental conditions, i.e. in the absence of apoptotic stimulus, *SNHG15* mainly affects the oxidation-related function of AIF. Given the full-length size of the AIF protein interacting with *SNNH15*, and the subcellular localization of the lncRNA, we speculate that *SNHG15* could interact with AIF upon the protein translation, coupling it to its correct translocation to the mitochondria. Interestingly, many studies have demonstrated that several members of the HSP70 family could bind to AIF and neutralize this protein [30]. Gurbuxani et al. showed that fragment between amino acids 150 to 228 of AIF is necessary for binding to HSP70 and this interaction block AIF nuclear localization [31]. As this fragment exists in both isoform of AIF, this process may occur for mitochondrial localization, so it is possible that binding *SNHG15* to AIF could prevent AIF neutralization by HSP70 family and help it to translocate to intermembrane space of mitochondria for contributing in respiratory chain activities and stress response. In agreement with our observations, previous studies have shown that *SNHG15* contribute to the molecular mechanism of cellular stress response. This lncRNA is a short-lived lncRNA (t1/2 < 4 h) and its expression level is increased significantly after 24 h cycloheximide used as a stressor, when its half-life was increased from 3.4 to more than 24 h after this treatment [32]. We showed that after depletion of *SNHG15*, ROS levels are significantly decreased, and drug sensitivity experiments showed that inhibition of *SNHG15* could sensitize CRC cells to 5-FU, which is a basic chemotherapeutic drug for CRC. Based on these results, we propose that the interaction between AIF and *SNHG15* may be help to incorporate this protein in ROS formation pathway.

Gene expression analysis after depletion of *SNHG15* revealed significant deregulation of multiple genes including *CTGF*, *GADD45A*, *GADD45B*, *HAS2*, *LAMC3*, *NRAS*, *BAG3*, *ERBB3*, *MYC* and *CASP3.* Most of these genes are known to play an important role in CRC tumor development and response to treatment. For example, *CTGF* decreases cell apoptosis and enhances CRC chemoresistance to 5-FU [33]. The inhibition of *GADD45A* leads to decrease in DNA repair and sensitize cells to ultraviolet-irradiation or cisplatin [34], while high level of its expression, turns CRC cells resistant to treatment with oxidative stress-inducing compounds [35]. *GADD45B* is significantly upregulated in CRC, and high levels of *GADD45B* expression are related to poor survival of patients [36]. Similarly, *HAS2* [37, 38], *NRAS* [39, 40] and *BAG3* [41–43] have well-established roles in CRC carcinogenesis and response to chemotherapy. Therefore, the gene expression changes linked to the loss of *SNHG15* are affecting central oncogenic pathways, including the response to chemotherapy and drug response, which is directly related of AIF function. However we cannot exclude that *SNHG15* has additional functions in the cell. For instance, *SNHG15* has been reported to control Slug stability in the nucleus [18] or regulate the levels of certain microRNAs [44–46]*.* It remains to be shown if the combination of other mechanisms with the AIF-dependent here described confers *SNHG15* its full pro-oncogenic activity.

Beyond CRC, the dysregulation and oncogenic role of *SNHG15* has been indicated in various types of cancer, including gastric [47] and hepatocellular carcinoma [48], osteosarcoma [44], as well as pancreatic [49] and breast cancers [46]. Our study, together with this body of work, including a recent study relating this lncRNA with increased liver metastasis of CRC tumors [50], indicate that *SNHG15* possesses a broad oncogenic activity, and suggests that the development of tools to target the lncRNA could have therapeutic value across multiple cancer types.

## Conclusion

Our results describe a role for the MYC-regulated *SNHG15* locus in colorectal cancer, role dependent on the lncRNA encoded by this bifunctional gene. The lncRNA *SNHG15* is able to promote colon cancer and mediating drug resistance, suggesting its potential as prognostic marker and target for RNA-based therapies.

## Additional files

Additional file 1: **Table S1.** Primers used for qRT-PCR. (DOCX 12 kb)

Additional file 2: **Table S2.** Guide RNAs used for CRISPR-Cas9 editing. (DOCX 11 kb)

Additional file 3: **Table S3.** The most significantly deregulated lncRNAs with high expression correlated with low survival of patients. (DOCX 13 kb)

Additional file 4: **Figure S1.** Association possibility of *SNHG15* to polysomes, *GAPDH* expression was evaluated as a positive control. (PNG 1830 kb)

Additional file 5: **Figure S2.** (A) Cell cycle analysis of CRC cells after synchronization with double thymidine block procedure. (B) *SNHG15* expression level of synchronized cells after each time point. Graphs shows mean ± SEM of values. (PNG 604 kb)

Additional file 6: **Table S4.** RNA seq analysis of LoVo cells after SNHG15 depletion.(XLSX 163 kb)

## Abbreviations

5-FU: Fluorouracil
AIF: Apoptosis Induced Factor
ATCC: American Type Culture Collection
BAG3: BCL2 Associated Athanogene 3
CASP3: Caspase 3
CCAT1: Colon Cancer Associated Transcript 1
ChIP: Chromatin immunoprecipitation
CHX: Cycloheximide
CL: Clone
CRC: Colorectal cancer
CRISPR: clustered regularly interspaced short palindromic repeats
CTGF: Connective Tissue Growth Factor
Ctrl: Control
DAPI: 4′,6-diamidino-2-phenylindole
DMEM: Dulbecco’s modified Eagle’s medium
EDTA: Ethylenediaminetetraacetic acid
ERBB3: Erb-B2 Receptor Tyrosine Kinase 3
FBS: Fetal bovine serum
FDR: False Discovery Rate
GADD45A: Growth Arrest and DNA Damage Inducible Alpha
GADD45B: Growth Arrest and DNA Damage Inducible Beta
GAPDH: Glyceraldehyde 3-phosphate dehydrogenase
GENCODE: Encyclopedia of DNA Elements
GFP: Green Fluorescent Protein
gRNAs: guide RNAs
HAS2: Hyaluronan Synthase 2
HOTAIR: HOX Transcript Antisense RNA
HPRT: Hypoxanthine Phosphoribosyltransferase
IPA: Ingenuty Pathway Analysis
LAMC3: Laminin Subunit Gamma 3
lncRNA: Long Noncoding RNA
MALAT1: Metastasis associated lung adenocarcinoma transcript 1
miRNAs: microRNA
MLS: Mitochondrial Localization Signal
MS: Mass Spectrometry
NC: Negative Control
ncRNAs: non-coding RNAs
PBS: Phosphate Buffered Saline
PFA: Paraformaldehyde
piRNAs: Piwi-interacting RNA
PTENP1: Phosphatase and Tensin Homolog Pseudogene 1
qRT-PCR: Quantitative Reverse Transcription Polymerase Chain Reaction
RFU: Relative Fluorescence Unit
RIP: RNA Immunoprecipitation
ROS: Reactive Oxygen Species
RPMI-1640: Roswell Park Memorial Institute
RT: Room Temperature
SD: Standard Deviation
SEM: Standard Error of the Mean
sgRNAs: single-guide RNAs
siRNA: Small interfering RNA
SNHG15: Small Nucleolar RNA Host Gene 15
SNORA9: Small Nucleolar RNA, H/ACA box 9
snoRNAs: Small Nucleolar RNA
TCGA: The Cancer Genome Atlas
TNM: Tumor, lymph node, metastasis
UCA1: Urothelial Cancer Associated 1
WT: Wild Type
