# Correction to: *SNHG15* is a bifunctional MYC-regulated noncoding locus encoding a lncRNA that promotes cell proliferation, invasion and drug resistance in colorectal cancer by interacting with AIF

**DOI:** 10.1186/s13046-019-1432-4

**Published:** 2019-10-31

**Authors:** Morvarid Saeinasab, Ahmad Reza Bahrami, Jovanna González, Francesco P. Marchese, Dannys Martinez, Seyed Javad Mowla, Maryam M. Matin, Maite Huarte

**Affiliations:** 1 Department of Biology, Faculty of Science, Ferdowsi University of Mashhad, Mashhad, Iran; 2 Industrial Biotechnology Reasearch Group, Institute of Biotechnology, Ferdowsi University of Mashhad, Mashhad, Iran; 3 Department of Gene Therapy and Regulation of Gene Expression, Center for Applied Medical Research, University of Navarra, Pamplona, Spain; 4 Institute of Health Research of Navarra (IdiSNA), Pamplona, Spain; 5 Department of Molecular Genetics, Faculty of Biological Sciences, Tarbiat Modares University, Tehran, Iran

**Correction to: J Exp Clin Cancer Res (2019) 38:172**

**https://doi.org/10.1186/s13046-019-1169-0**

In the original publication of this article,[1] the Funding section needs to be revised, and the corrected Funding section appears below. We apologize for any confusion this may have caused.

## Funding

This work has been supported by FEDER/Ministerio de Ciencia, Innovación y Universidades - Agencia Estatal de Investigación/ BFU2017–82773-P. This study was part of a PhD dissertation and was supported by Ferdowsi University of Mashhad (No. 41615).
