# Supplementary material for: SNHG15 is a bifunctional MYC-regulated noncoding locus encoding a lncRNA that promotes cell proliferation, invasion and drug resistance in colorectal cancer by interacting with AIF
Source: J Exp Clin Cancer Res. 2019 Apr 24;38:172. doi: 10.1186/s13046-019-1169-0 (PMC6480895; doi:10.1186/s13046-019-1169-0)
Supplement: Supplementary file 1 — Table S1. Primers used for qRT-PCR. (DOCX 12 kb) [file 13046_2019_1169_MOESM1_ESM.docx]

**Table S1:** Primers used for qRT-PCR

| **Name** | **Forward Primer (5'->3')** | **Reverse Primer (5'->3')** |
| --- | --- | --- |
| SNHG15-PP1 | CACAAGAGTGCCTGCCATC | GGCAGCCACTGAAGGTATC |
| SNHG15-PP2 | GTTGCCTGACCATTCCTGAG | AGTAAGCTCTTCCACTTTGAGACC |
| SNORA9 | GCATTCCTTCAGTGCTTGCT | ATTGTCTGAAATTTCTATAACCTTGCT |
| MYC | CACCAGCAGCGACTCTGA' | GATCCAGACTCTGACCTTTTGC |
| CTGF | AGCTGACCTGGAAGAGAACATT | GCTCGGTATGTCTTCATGCTG |
| GADD45A | GCAGTTTGCAATATGACTTTGG | CATCCCCCACCTTATCCAT |
| GADD45B | CGGCCAAGTTGATGAATGT | GATTTGCAGGGCGATGTC |
| NRAS | AATACATGAGGACAGGCGAAG | GAGTCTTTTACTCGCTTAATCTGCTC |
| CASP | TGTGGAATTGATGCGTGATG | TCCAAAAATTATTCCTTCTTCACC |
| LAMC3 | CCTGTACGACCATCCCAGAG | GCCATCATCACAGACCTCAC |
| HAS2 | TTATTACCTCAATTTTGGAAACTGC | TCAGGATACATAGAAACCTCTCACAA |
| BAG3 | GCACCACTACGTGGAACGA | GGTGGCCTTCCCTAGCAG |
| ERBB3 | TCGAGCAACATTGATGGATTT | GATCTTGTGCCAGGGGTCT |
| MALAT1 | GACGGAGGTTGAGATGAAGC | ATTCGGGGCTCTGTAGTCCT |
| U6 | GCTTGCTTCAGCACATA | AAAAACATGGAACTCTTCACG |
| HPRT | TGACACTGGCAAAACAATGCA | GGTCCTTTTCACCAGCAAGCT |
| GAPDH | AGCCACATCGCTCAGACAC | GCCCAATACGACCAAATCC |
