# Supplementary material for: SNHG15 is a bifunctional MYC-regulated noncoding locus encoding a lncRNA that promotes cell proliferation, invasion and drug resistance in colorectal cancer by interacting with AIF
Source: J Exp Clin Cancer Res. 2019 Apr 24;38:172. doi: 10.1186/s13046-019-1169-0 (PMC6480895; doi:10.1186/s13046-019-1169-0)
Supplement: Supplementary file 2 — Table S2. Guide RNAs used for CRISPR-Cas9 editing. (DOCX 11 kb) [file 13046_2019_1169_MOESM2_ESM.docx]

**Table S2**. Guide RNAs used for CRISPR-Cas9 editing

| **Name** | **sequence 5'->3'** |
| --- | --- |
| SNHG15 sgRNA-1.1 | CACCGAAGACCCTGCGTCTTCTTGA |
| SNHG15 sgRNA-1.2 | AAACTCAAGAAGACGCAGGGTCTTC |
| SNHG15 sgRNA-2.1 | CACCGCCTGTGTTCCTCTGGGTGGT |
| SNHG15 sgRNA-2.2 | AAACACCACCCAGAGGAACACAGGC |
