# Supplementary material for: SNHG15 is a bifunctional MYC-regulated noncoding locus encoding a lncRNA that promotes cell proliferation, invasion and drug resistance in colorectal cancer by interacting with AIF
Source: J Exp Clin Cancer Res. 2019 Apr 24;38:172. doi: 10.1186/s13046-019-1169-0 (PMC6480895; doi:10.1186/s13046-019-1169-0)
Supplement: Supplementary file 3 — Table S3. The most significantly deregulated lncRNAs with high expression correlated with low survival of patients. (DOCX 13 kb) [file 13046_2019_1169_MOESM3_ESM.docx]

**Table S3.** The most significantly deregulated lncRNAs with high expression correlated with low survival of patients

| **Name** | **Gencode Gene ID** | **Chromosome** | **Position** | **p value** | **p value of survival** |
| --- | --- | --- | --- | --- | --- |
| *SNHG15* | ENSG00000232956.8 | 7 | 44,983,023-44,986,834 | 7.5e-23 | 4e−05 |
| *RP11−775H9.2* | ENSG00000251567.5 | 4 | 136,173,199-136,395,471 | 1.02e-07 | 0.00011 |
| *RP11−369K17.1* | ENSG00000253619.1 | 8 | 120,913,065-121,119,754 | 1.51e-07 | 0.00014 |
| *RP11−774D14.1* | ENSG00000251629.6 | 5 | 20,611,840-20,937,691 | 1.36e-06 | 0.00022 |
| *RP11−739G5.1* | ENSG00000258631.2 | 15 | 93,589,867-93,760,799 | 1.52e-10 | 0.00032 |
| *RP1−154K9.2* | ENSG00000231772.5 | X | 42,252,459-42,699,291 | 6.26e-09 | 0.00046 |
| *RP11−341G5.1* | ENST00000510907.5 | 4 | 13,655,179-13,931,228 | 3.04e-08 | 0.00143 |
| *RP1−15D23.2* | ENSG00000224228.2 | 1 | 172,775,905-173,064,015 | 1.62e-07 | 0.0019 |
| *RP11−31F15.2* | ENSG00000238198.1 | 1 | 113,011,687-113,073,105 | 8.57e-11 | 0.00192 |
| *RP11−217C7.1* | ENSG00000249675.1 | 4 | 166,388,701-166,526,119 | 3.07e-13 | 0.00246 |
| *AC004158.2* | ENSG00000261008.6 | 16 | 72,283,301-72,664,947 | 7.20e-14 | 0.00282 |
| *RP11−545D19.1* | ENSG00000233571.1 | X | 34,206,725-34,415,537 | 2.22e-09 | 0.00601 |
| *RP11−260O18.1* | ENSG00000239440.5 | 3 | 81,986,138-82,463,675 | 1.09e-06 | 0.00938 |
| *RP11−648K4.2* | ENSG00000259560.1 | 15 | 87,432,058-87,703,852 | 2.26e-10 | 0.00983 |
