# Supplementary figures and images for: SNHG15 is a bifunctional MYC-regulated noncoding locus encoding a lncRNA that promotes cell proliferation, invasion and drug resistance in colorectal cancer by interacting with AIF
Source: J Exp Clin Cancer Res. 2019 Apr 24;38:172. doi: 10.1186/s13046-019-1169-0 (PMC6480895; doi:10.1186/s13046-019-1169-0)

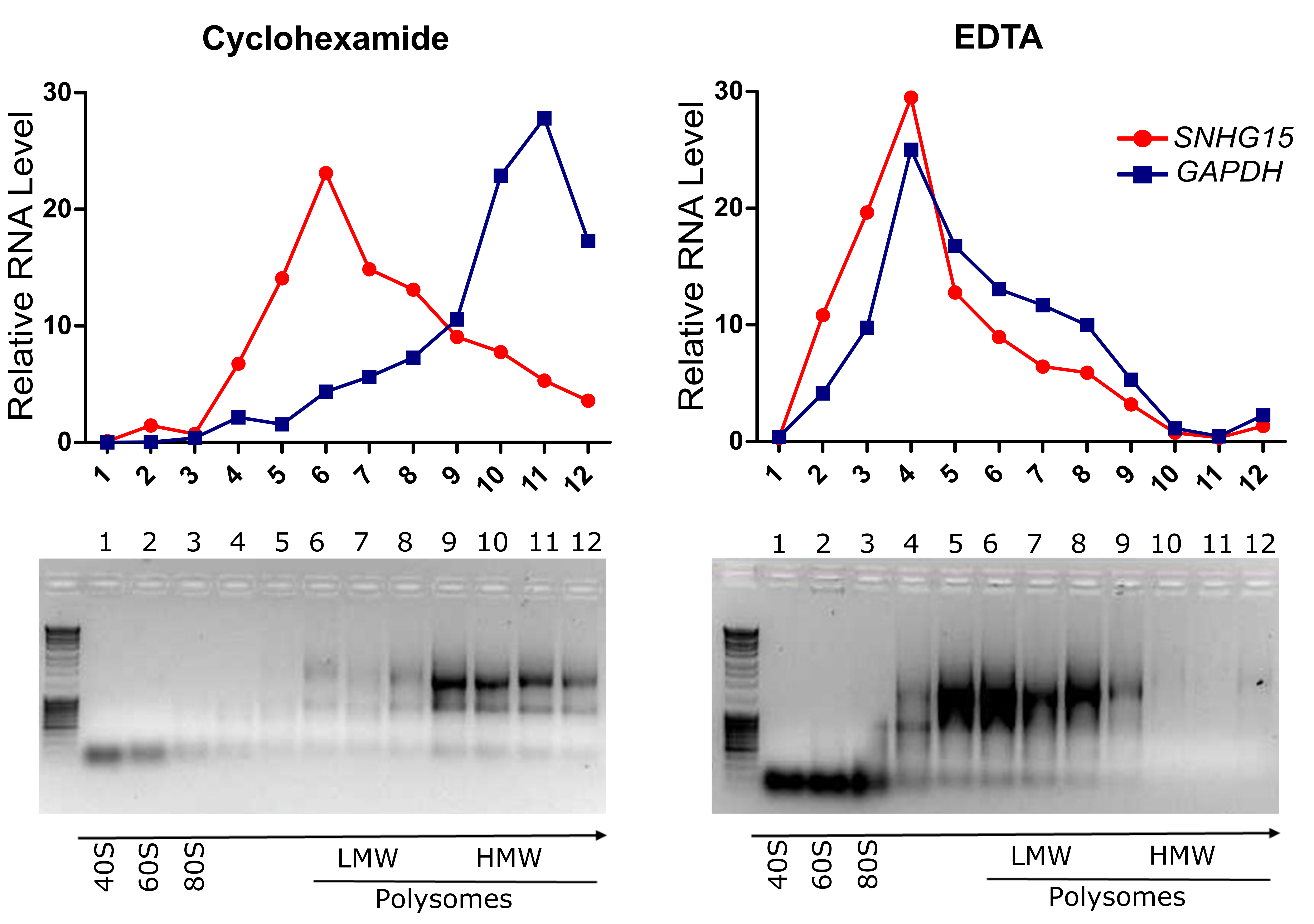

Supplement: Supplementary file 4 — Figure S1. Association possibility of SNHG15 to polysomes, GAPDH expression was evaluated as a positive control. (PNG 1830 kb) [file 13046_2019_1169_MOESM4_ESM.png]

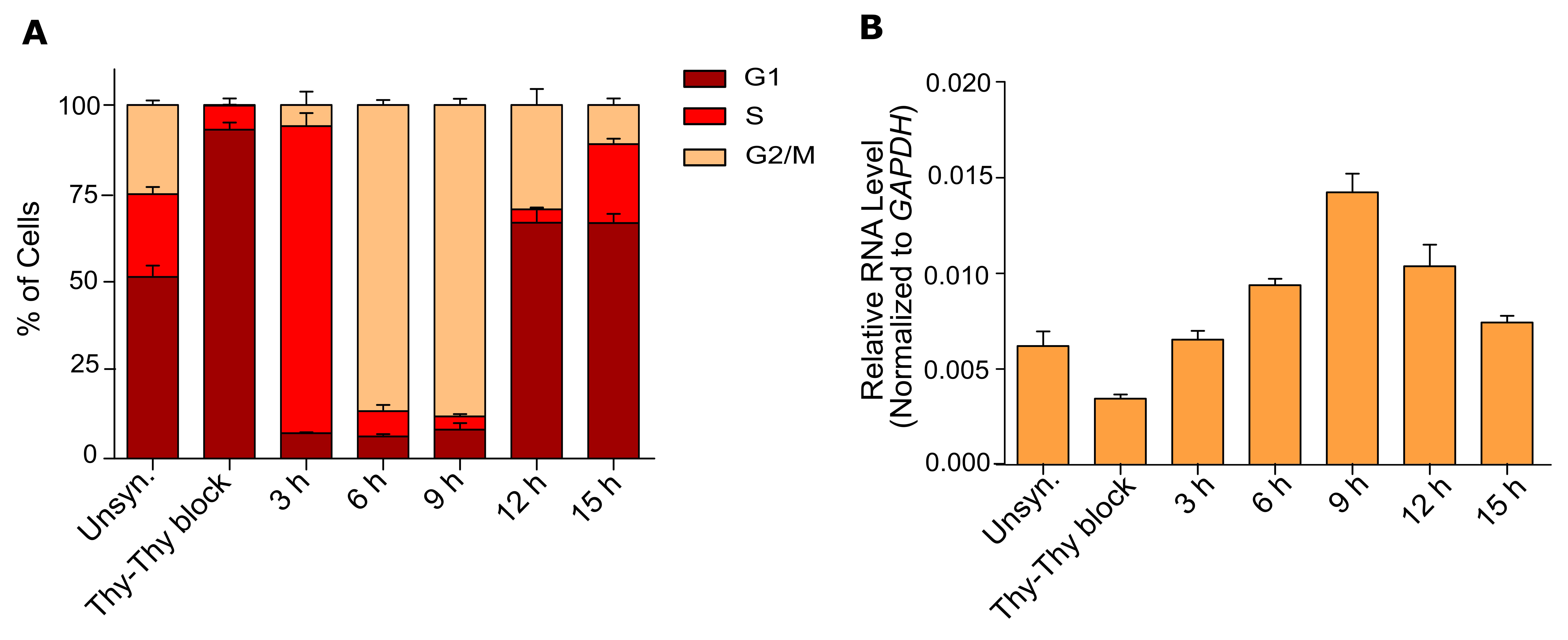

Supplement: Supplementary file 5 — Figure S2. (A) Cell cycle analysis of CRC cells after synchronization with double thymidine block procedure. (B) SNHG15 expression level of synchronized cells after each time point. Graphs shows mean ± SEM of values. (PNG 604 kb) [file 13046_2019_1169_MOESM5_ESM.png]
